# Expression of FGD3 gene as prognostic factor in young breast cancer patients

**DOI:** 10.1038/s41598-019-51766-w

**Published:** 2019-10-23

**Authors:** Irene Renda, Simonetta Bianchi, Vania Vezzosi, Jacopo Nori, Ermanno Vanzi, Ketty Tavella, Tommaso Susini

**Affiliations:** 1 Breast Unit, Gynecology Section, Department of Health Sciences, University of Florence, Florence, Italy; 2 Pathology Unit, Department of Health Sciences, University of Florence, Florence, Italy; 3 Diagnostic Senology Unit, Azienda Ospedaliero-Universitaria Careggi, Florence, Italy; 4 Medical Oncology Unit, Azienda Ospedaliero-Universitaria Careggi, Florence, Italy

**Keywords:** Risk factors, Breast cancer

## Abstract

The FGD3 gene works as a cell migration inhibitor and seems to be a promising indicator of outcome in some human cancers including breast. In this study, we analysed for the first time the prognostic role of FGD3 in young breast cancer patients. We studied the relationship between traditional prognostic factors, FGD3 expression and outcome in ≤40 years breast cancer patients. We found that lower FGD3 expression decreased the probability of disease-free survival (*p* = 0.042) and overall survival (*p* = 0.007). In a multivariate analysis for overall survival AJCC stage (*p* = 0.005) and FGD3 expression (*p* = 0.03) resulted independent prognostic factors. Low FGD3 expression increased the risk of death from disease (HR 5.73, *p* = 0.03). Moreover, low FGD3 expression was associated with more widespread lymph node involvement (*p* = 0.04) and a lower FGD3 staining intensity was found in positive-lymph-node patients vs negative (*p* = 0.003) and in patients with ≥10 involved lymph nodes vs <10 (*p* = 0.05). Our results suggest FGD3 to be a significant independent prognostic factor in young breast cancer patients in terms of disease-free survival and overall survival. A lower expression increased the risk of recurrence and death from disease and was associated with widespread lymph node metastases.

## Introduction

Breast cancer is the most common cancer in women. Mortality is decreasing thanks to earlier detection and better adjuvant and neoadjuvant treatment, despite incidence is increasing. Only 5–7% of breast cancers occur in ≤40-year-old women, except for Latin American women, who present a 15% figure^1,2^, but incidence is increasing in this group too. Young women have generally more aggressive tumours than older women do. They present tumours of higher grade, larger size, positive lymph nodes^3^, a higher proportion of basal-like, HER-2 positive tumours and a lower proportion of luminal-A tumours^4^. As a consequence, young women also present a higher risk of recurrence and death^5^. Although several studies have showed the higher tumour aggressiveness in young women, they have not demonstrated the regulatory mechanism, yet. There is a clear need of new prognostic factors to better stratify patients, to predict individual outcome among the same histological and molecular subgroup. For this reason, a number of researches are being carried out to identify new prognostic factors in breast cancer patients at all ages^6^.

One of the most promising new prognostic factors is the expression of FGD3 gene (Facio-Genital Dysplasia 3 gene), localised on long arm of chromosome 9 (Chr9q22.31), codifying for FYVE, RhoGEF and PH-Domain containing protein 3. It has been identified by Hayakawa *et al*. as a guanine nucleotide exchange factor that targets cell division control protein 42 (CDC42), inducing its activation and modifying cell morphology with formation of lamellipodia^7^. As a consequence, it plays an inhibiting role on cell migration in both normal and neoplastic cells^7^. Therefore, a lower FGD3 expression seems to indicate a major risk of cell migration, whereas a higher expression seems to indicate a minor risk^8^. The role of this gene was first identified in 2013 during the Sage Bionetwork/DREAM Breast Cancer Prognosis Challenge (BCC), a crowdsourced research study by Margolin *et al*.^9^, realized to identify genes associated with prognosis in breast cancer, using a data set of 1981 cancer samples (METABRIC). Cheng *et al*. found that FGD3 gene was the top-ranked protective gene for breast cancer and that silencing FGD3 gene resulted in silencing the adjacent SUSD3 gene, the second top-ranked protective gene in the study^10,11^. A further study by Yang *et al*. in 2014^12^ analysed the use of a new prognostic test (BCAM test), composed of FGD3-SUSD3 metagene, other seven attractor metagenes (CIN, MES, LYM, END, CD68, DNAJB9 and CXCL12), tumour size and positive lymph nodes number. BCAM test was universally applicable in all tumour stages and subtypes. The low expression of FGD3-SUSD3 metagene was found to be more associated with poor outcome than the lack of ESR1 expression^10^. A recent study by Willis *et al*.^13^ has demonstrated the prognostic role of FGD3 expression in a large cohort of breast cancer patients, compared with other important genes associated with proliferation as MKI67^14^, PCNA^15^ and AURKA^16^, regardless of ER status and molecular subtype. Lower expression was associated with a higher rate of lymph-node involvement and with decreased disease-free survival and overall survival. They also demonstrated its prognostic value in head and neck squamous cell carcinoma, lung adenocarcinoma, cervical squamous cell carcinoma, bladder urothelial carcinoma and sarcoma. No studies so far have specifically investigated the role of FGD3 in young patients with breast cancer.

The current study was undertaken to test the prognostic value of FGD3 expression in a young breast cancer population. For this purpose, we analysed and compared the prognostic significance of traditional prognostic factors and FGD3 expression in our series of young breast cancer patients in terms of disease-free survival (DFS) and overall survival (OS).

## Results

### Descriptive characteristics

**Figure 1:**
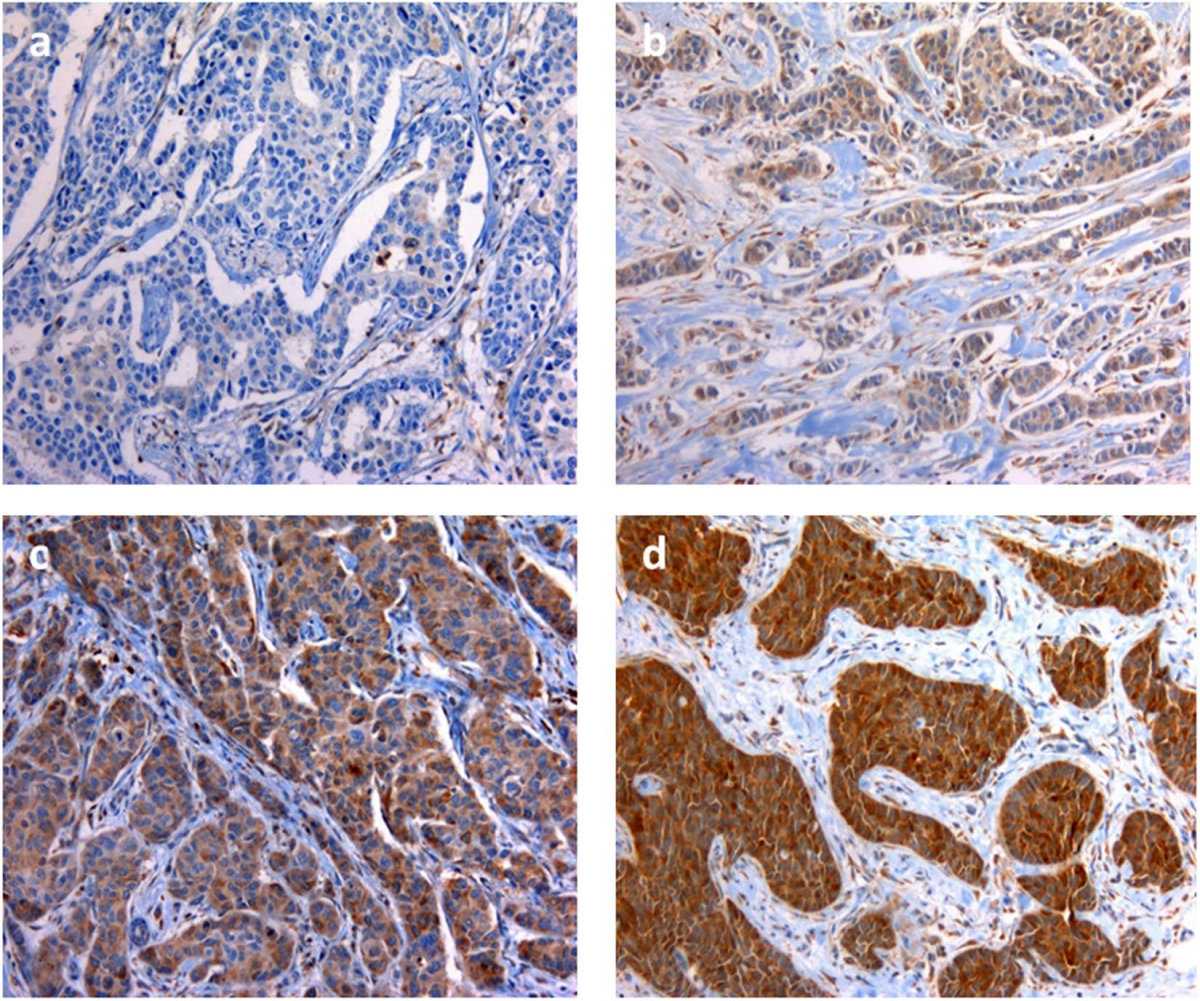
Immunostaining for FGD3 in breast cancer tissue blocks (x400). Note: Staining was (**a**) undetectable (−), (**b**) low (+), (**c**) moderate (++), (**d**) intense (+++).

We analysed 60 patients, with an average age of 37.0 (range 21–40 years). FGD3 expression was assessed by immunohistochemistry (Fig. 1). Distribution according to FGD3 expression by clinical and pathologic characteristics of patients showed no significant difference, except for number of patients who underwent to axillary lymphadenectomy, that were significantly more frequent among patients with low FGD3 expression. Data are summarised in Table 1.

**Table 1:** Clinical and pathologic characteristics of patients: overall and according to FGD3 expression.

Characteristic	All	%	FGD3−	%	FGD3+	%	*p*
Age, median (range)	37 (21–40)	—	36 (21–40)	—	37 (26–40)	—	—
Grade							
G1	6	10.5	—	—	6	10.5	0.14
G2	18	31.6	6	10.5	12	21.1	
G3	33	57.9	14	24.6	19	33.3	
Total	57	100.0	20	35.1	37	64.9	
LVSI							
Yes	32	53.3	8	13.3	20	33.3	0.42
No	28	46.7	13	21.7	19	31.7	
Total	60	100.0	21	35.0	39	65.0	
**Molecular Subtype**							
Luminal A	29	59.2	8	16.4	21	42.9	0.78
Luminal B	9	18.4	4	8.2	5	10.2	
Her2+	3	6.1	1	2.0	2	4.1	
Triple negative	8	16.3	2	2.0	6	12.2	
Total	49	100.0	15	30.6	34	69.4	
**Ki67**							
<15%	14	26.9	6	11.5	8	15.4	0.51
≥15%	38	73.1	11	21.2	27	51.9	
Total	52	100.0	17	32.7	35	67.3	
**AJCC stage**							
I	26	43.3	7	11.7	19	31.7	0.16
II	15	25.0	6	10.0	9	15.0	
III	12	20.0	3	5.0	9	15.0	
IV	7	11.7	5	8.0	2	3.3	
Total	60	100.0	21	35.0	39	65.0	
**Primary tumour surgery**							
BCS	31	51.7	12	20.0	19	31.7	0.60
Mastectomy	29	48.3	9	15.0	20	33.3	
Total	60	100.0	21	35.0	39	65.0	
**Axillary lymph node surgery**							
SLB neg	20	33.3	3	5.0	17	28.3	0.02
SLB pos + AD	40	66.7	18	30.0	22	36.7	
Total	60	100.0	21	35.0	39	65.0	
**Neoadjuvant Chemotherapy**							
Yes	19	31.7	8	13.3	11	18.3	0.56
No	41	68.3	13	21.7	28	46.7	
Total	60	100.0	21	35.0	39	65.0	
**Adjuvant Chemotherapy**							
Yes	39	65.0	13	21.7	26	43.3	0.78
No	21	35.0	8	13.3	13	21.7	
Total	60	100.0	21	35.0	39	65.0	
**Hormonotherapy**							
Yes	38	63.3	15	25.0	23	38.3	0.41
No	22	36.7	6	10.0	16	26.7	
Total	60	100.0	21	35.0	39	65.0	
**Trastuzumab**							
Yes	10	16.7	4	6.7	6	10.0	0.73
No	50	83.3	17	28.3	33	55.0	
Total	60	100.0	21	35.0	39	65.0	
**Adjuvant Radiotherapy**							
Yes	37	61.7	14	23.3	23	38.3	0.59
No	23	38.3	7	11.7	16	26.7	
Total	60	100.0	21	35.0	39	65.0	

Abbreviations: LVSI, lympho-vascular space invasion; AJCC stage, The American Joint Committee on Cancer staging; BCS, breast conservative surgery; SLB, sentinel lymph node biopsy; AD, axillary dissection.

During the study period, 26 patients (43.3%) experienced recurrence and 10 patients (16.7%) died for the disease.

### Association between traditional prognostic factors and outcome

We analysed the association of the most relevant established prognostic factors for breast cancer with outcome using Cox proportional hazards regression analysis. Grade (*p* = 0.06 on OS), pT stage (*p* < 0.001 on both DFS and OS), pN stage (*p* = 0.02 on OS), AJCC stage (*p* = 0.08 on DFS, *p* = 0.001 on OS) and Ki67 (*p* = 0.09 on DFS) have confirmed to be significant prognostic factors or to approach the significance (data not shown).

### Association between FGD3 expression and outcome

**Figure 2:**
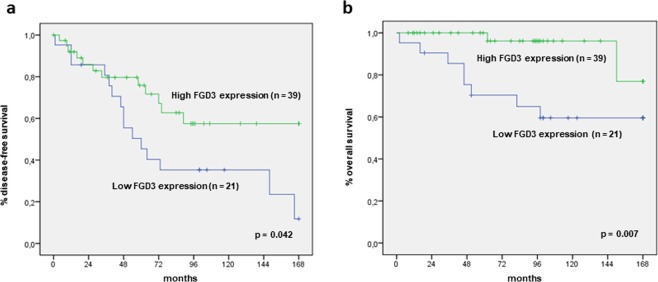
Disease-free survival and overall survival according to FGD3 expression. Note: (**a**) Disease-free survival; (**b**) Overall survival.

Concerning FGD3, among 60 analysed samples, 21 (35%) showed low FGD3 expression and 39 (65%) showed high FGD3 expression. To compare the distribution of recurrence and death from disease between the two groups, a Fisher’s Exact Test was carried out (Table 2). Recurrence and death from disease were significantly higher among patients with low FGD3 expression, (*p* = 0.003 and *p* = 0.001, respectively). Moreover, using KM method, we found that patients with low FGD3 expressing tumours had significantly reduced DFS (*p* = 0.042) and OS (*p* = 0.007) (Fig. 2). In addition, we evaluated the impact of FGD3 expression on outcome according to year of diagnosis, comparing patients diagnosed before or after 2008, to exclude the possible interference on prognosis due to more effective treatments in the latter period. High-FGD3-expressing patients displayed significantly more favourable outcome in both cohorts of patients (data not shown).

**Table 2:** Association between FGD3 expression and recurrence/death from disease.

FGD3 Expression	Recurrence	Total	%	*p*	Death from disease	Total	%	*p*
Low (0% - or ≤30%+)	15	21	71.4	0.003	8	21	38.1	0.001
High (>30%+or ++ or +++)	12	39	30.8		2	39	5.1	
Total	27	60	100.0		10	60	100.0

### Multivariate analysis

Table 3 shows the results of multivariate Cox proportional hazards regression analysis in which AJCC stage (I-II vs III-IV), FGD3 expression and tumour grade were tested simultaneously to assess the risk ratio for death from disease and the independence of each variable. A higher AJCC stage (III-IV) was the strongest independent predictor of poor outcome (HR = 9.36, *p* = 0.005). The second stronger independent predictor of poor outcome was FGD3 expression (HR = 5.73, *p* = 0.03). On the contrary, tumour grade was not a significant predictor of prognosis by multivariate analysis.

**Table 3:** Multivariate Cox analysis for death from disease.

Parameters		Overall survival		
		HR	95% CI	*P*
AJCC stage	**I-II**	Ref.		
	**III-IV**	9.36	1.98–44.22	0.005
FGD3 expression	**High**	Ref.		
	**Low**	5.73	1.20–27.32	0.03
Grading	**G1-G2**	Ref.		
	**G3**	2.76	0.58–13.21	0.2

### Association between FGD3 expression and outcome stratified by AJCC stage and molecular subtype

**Figure 3:**
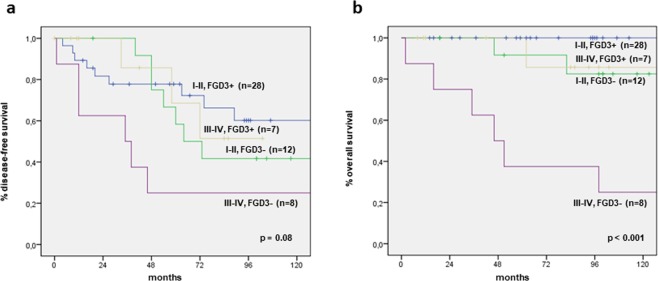
Disease-free survival and overall survival according to FGD3 expression in AJCC stage stratified groups. Note: (**a**) Disease-free survival; (**b**) Overall survival. Abbreviations: I-II, FGD3+ = I-II AJCC stage with high FGD3 expression; I-II, FGD3− = I-II AJCC stage with low FGD3 expression; III-IV, FGD3+ = III-IV AJCC stage with high FGD3 expression; III-IV, FGD3− = III-IV AJCC stage with low FGD3 expression.

We stratified our population by AJCC stage, identifying patients with low AJCC stage (I-II) and high AJCC stage (III-IV), and we analysed the association of FGD3 expression with outcome in the two groups by Kaplan-Meier method, as shown in Fig. 3. In terms of OS, patients with stage I-II AJCC and high FGD3 expression were all alive at 10 years of follow-up, while 10 of the 13 patients with stage I-II and low FGD3 expression (76.9%) were alive at 10 years. Patients with stage III-IV AJCC and high FGD3 expression had a quite better prognosis than those with low stage and low FGD expression and a much better prognosis than patients with stage III-IV AJCC and low FGD3 expression (*p* < 0.001). The significance was only approached in terms of DFS (*p* = 0.08), but in each AJCC stage group, patients with high FGD3 expression had longer DFS at 10 years than those with low FGD3 expression. Again, high-FGD3-expressing patients with stage III-IV had a better outcome compared to low-FGD3-expressing patients with stage I-II. Similarly, we stratified our population by molecular subtype and we analysed the association of FGD3 expression with outcome in each subtype. Again, high-FGD3-expressing patients had a more favourable outcome, both in terms of DFS and OS (*p* = 0.018 and *p* < 0.001, respectively) (data not shown). In addition, we adjusted the analysis by AJCC stage and immunohistochemical subtype and found that FGD3 expression retained its significance (*p* = 0.02 and *p* = 0.006, respectively).

### Association between FGD3 expression and lymph node involvement

We analysed FGD3 expression and lymph node involvement using Fisher’s Exact Test (Table 4). We found that low FGD3 expressing patients had higher incidence of lymph node metastases as compared with high FGD3 expressing ones, (61.9% versus 41.0%), but the difference was not significant (*p* = 0.12). However, among patients with low FGD3 expression there was a significantly higher incidence of massive lymph nodal spread (≥10 positive lymph nodes) (61.5% versus 25.0%; *p* = 0.04). Finally, we analysed the distribution of FGD3 expression staining intensity (from – to + + + ) in lymph node negative patients (n = 31) and lymph node positive ones (n = 29) using a Chi Square Test: the distribution was significantly different (*p = *0.007) and there was an increasing rate of positive lymph nodes as FGD3 staining intensity decreased, using Chi Square Test for Trend (*p* = 0.003). Similarly, among patients with positive lymph nodes, there was a difference in distribution of FGD3 staining intensity in patients with <10 positive lymph nodes (n = 22) and ≥10 positive lymph nodes (n = 12) (*p* = 0.05) and a Chi Square Test for Trend revealed an increasing in lymph node number involvement as FGD3 staining intensity decreased (*p* = 0.01) (data not shown).

**Table 4:** Association between FGD3 expression and lymph node involvement.

FGD3 Expression	N +	Total	%	*p*	pN+ ≥ 10	Total	%	*p*
Low (0% - or ≤30%+)	13	21	61.9	0.12	8	13	61.5	0.04
High (>30%+ or ++ or +++)	16	39	41.0		4	16	25.0	
Total	29	60	100.0		12	29	100.0	

## Discussion

We found a high prevalence of classical predictors of poor outcome in our series of young breast cancer patients. So, our findings confirmed the increased breast cancer aggressiveness in young patients as compared to general population. Hence, more than half of patients had G3, multifocal/multicentric tumours with lymphovascular space invasion. One third of tumours was >2 cm at diagnosis (≥pT2 stage). Nearly half of patients had positive lymph nodes, with a half of them having >3 involved lymph nodes (pN2 - pN3). Metastases at diagnosis were present in a relatively high percentage of cases. These figures were in accordance with a study by Gnerlich *et al*.^3^ reporting higher percentages of G3 tumours (56% vs 26%), >2 cm lesions and involved lymph nodes (38% vs 25%) among young women. Compared to general population, in the current series we found a higher percentage of Her2 + tumours (24.5% vs 13–15%^1^) and triple negative tumours (16.3% vs 11.2%^17^), whereas the incidence of ER/PgR + tumours was similar (77.6% vs 80%). Our findings are in accordance to Anders *et al*.^17^ study, which showed a higher prevalence of Her2 + (52% vs 24%) and triple negative tumours (7% vs 2.6%) among young women compared to older women, but differed from it about the incidence of ER/PgR + tumours, which resulted lower in younger patients compared to older ones in Anders *et al*.’s study (71% vs 80%). As a consequence of multicentric and large size disease, in our series there was a higher percentage of mastectomy (48.3%) and a lower percentage of conservative operations (51.7%), compared to older women in whom percentages are 14% vs 86%, respectively^18^. This result shows that in clinical practice there is a frequent recourse to mastectomy in a group of patients in which we would theoretically prefer to use conservative operations, to preserve psychological and functional aspects.

Traditional prognostic factors confirmed their influence on outcome in the current series. According to literature, the significance was achieved by tumour grade^19^ on OS, pT stage^20^ on DFS and OS, pN stage^20^ on OS, AJCC stage on OS. The failure to reach significance of universally approved prognostic factors in some subgroup analysis was probably due to the limited number of cases.

The most important finding of our study was the prognostic role of FGD3 expression on both DFS and OS, despite the relatively small number of cases, in accordance with previous studies who analysed patients not selected by age^9,10,12,13^. Concerning the choice of the cut-off for analysis of IHC FGD3 expression, a similar approach has been used in several immunohistochemical studies^21,22^. Moreover, the chosen cut-off point seems to be in accordance with Willis *et al*.’s one, as they talked about “high expression” and not about percentage of positive cells or staining intensity only^13^. We found that low FGD3 expression was associated with recurrence: patients with low FGD3 expression relapsed significantly more frequently than those with high FGD3 expression. Moreover, patients with low FGD3 expression had a significantly higher percentage of death from disease. Thus, we found that low FGD3 expression had a significant negative effect on disease-free survival and overall survival. Our result is in accordance with the FGD3 gene inhibiting role on cell migration, which was demonstrated in other studies^7^. Interestingly, our results indicate that FGD3 expression represents an independent predictor of clinical outcome in young breast cancer patients in terms of OS, second only to high AJCC stage (III-IV), according to literature^23^. Whereas, a well-known risk factor such as tumour grade, was not found to be significant and independent. This is an interesting finding, considering the poorer outcome of young breast cancer patients and the failure in identifying this higher risk subgroup by traditional predictors. Indeed, the introduction of new prognostic factors such as FGD3 expression could help selecting patients at higher risk. This could influence the choice of individual treatment and possibly translate into improved outcome.

Another relevant finding was that stratification by FGD3 expression within two subgroups of different AJCC stage^23^ (stage I-II AJCC vs stage III-IV AJCC) identified patients with different outcome within the same AJCC stage subgroup. In particular, we found that low FGD3 expression significantly worsen the prognosis of patients in the same AJCC stage. Furthermore, according to our results, it seems that FGD3 expression exceeds the strength of AJCC stage in determining patients’ outcome. In fact, patients with advanced disease (stage III-IV AJCC) and high-FGD3-expression did better than patients with early stage disease (stage I-II AJCC) but low-FGD3-expression (Fig. 3). This result suggests that biological features of the tumour such as FGD3 expression may represent an even stronger determinant of survival than the classical tumour stage.

Previous studies^7,13^ pointed out the inhibiting role of FGD3 on cellular migration and its influence in lymph node metastases. Therefore, we studied the association between FGD3 expression and lymph node involvement in our series. We found that lower FGD3 expression was significantly associated with a more widespread lymph node involvement (Table 4). Moreover, FGD3 staining intensity was significantly higher in patients without lymph node involvement compared to those with involved lymph nodes. Conversely, the FGD3 staining intensity among patients with ≥10 positive lymph nodes was significantly lower than that of patients with <10 positive lymph nodes. This finding further suggests an association between lower FGD3 expression and lymph node involvement, in accordance with previous studies^7,13^. So, the evaluation of FGD3 expression could be helpful to estimate the risk of lymph node involvement in patients without clinical evidence of metastasis.

Willis *et al*.^13^ have recently demonstrated by RT-qPCR that estradiol stimulation increases FGD3 mRNA expression level, through the ESR1 binding site within the gene. This interesting finding may introduce the hypothesis of a potential role for FGD3 as a therapeutic target, in addition to its prognostic role. Hence, the Collaborative Group on Hormonal Factors in Breast Cancer reported in two different studies that both oral contraceptive^24^ and hormonal replacement therapy^25^ increased the risk of breast cancer due to oestrogen stimulation. On the other hand, these same studies observed that oestrogen-induced breast cancers were less aggressive than general population’s breast cancers. Therefore, we might now hypothesize that FGD3 may have a role in this mechanism: oestrogen stimulation could increase FGD3 expression, which in turn may determine a better prognosis in oestrogen treated patients. Our results seems to confirm that high FGD3 expression is a protective factor against recurrence and death from disease. Considering the relatively small number of patients in this study, our results must be interpreted with caution. Therefore, further studies on FGD3 in breast cancer are warranted to evaluate both its prognostic significance and to explore the possibility of modulating this gene expression for therapeutic purposes.

The current study confirmed the higher breast cancer aggressiveness in younger women. We found that FGD3 expression was a significant and independent predictor of clinical outcome. In particular, high FGD3 expression was a protective factor against recurrence, death from disease and lymph node involvement. FGD3 expression allowed to distinguish patients with significantly different clinical fate within the same AJCC stage, with the role of FGD3 possibly being stronger than that of AJCC stage in determining outcome. Our findings in young women are in line with those of previous studies in breast cancers not selected by age^9,10,12,13^. To our knowledge, this is the first study to investigate the role of FGD3 expression using a simple and inexpensive method such as IHC and reporting significant differences in clinical outcome even with a relatively small number of patients. This represents an example of the possible usefulness of introducing FGD3 assessment at the clinical level to translate the advances from previous studies on very large cohorts of patients and using more sophisticated techniques in the everyday practice of a single institution.

## Methods

### Patients selection and data collection

We identified 60 ≤ 40-year-old women suffering from breast cancer, surgically treated and followed between 1998 and 2018 at the Breast Unit of the Gynaecology and Obstetrics Department, Careggi Hospital, University of Florence. We collected patients’ data from medical records, including surgical treatment of primary tumour and axillary lymph nodes, neoadjuvant and adjuvant treatment, clinical and pathological characteristics of tumours, disease-free survival and overall survival. After surgery, patients had follow up visits every 6 months during the first 5 years and every year, thereafter. Mammogram and ultrasound scan of the breast were performed every 12 months. Average follow up interval was 92.5 months (range 8–168 months). All patients gave their written informed consent to the use of tissue blocks for the study purposes. The study was approved by the local ethics committee (Careggi Hospital, University of Florence).

### FGD3 expression

Immunohistochemical evaluation of FGD3 expression was performed on slides hours to days after deep sectioning of formalin-fixed, paraffin embedded tissue blocks. Slides were stored at 4 °C in order to test possible antigen recovery. In addition, antigen preservation was verified prior to FGD3 analysis by immunohistochemistry with internal positive anatomic controls. The use of this procedure prevented proteolytic degradation of the samples, because it is known that formalin-fixed tissue within paraffin blocks maintain intact protein structures for even more than 20 years, in contrast with old slides in which proteolytic degradation may occur after some years. FGD3 protein expression was evaluated using a rabbit polyclonal antibody against FGD3 at a dilution of 1:750 (Sigma-Aldrich, St. Louis, MO, Cat# SAB1401929, RRID:AB_10609712). Paraffin removing, antigen recovery and antibody incubation were carried out using Bench Mark Ultra device, according to a set protocol. The protein expression was evaluated using the detection system HRP ultra View Universal DAB Detection Kit (Ventana, Tucson, Arizona). Positive control was obtained using tonsillar tissue. Negative control was obtained using a rabbit serum antibody (Normal, Dako Agilent, Carpinteria, CA, Cat# A020602, RRID:AB_578507). The results were expressed as percentage of positive cells and as staining intensity (undetectable−, weak+, moderate++, strong+++), as shown in Fig. 1.

### Cellular reactivity cut-off point

To evaluate the prognostic value of FGD3 expression, we compared the patients’ disease-free survival and overall survival after dividing them into two groups according to different cut-off points. The P values were significant for DFS (*p* = 0.04) and OS (*p* = 0.007) when we arbitrarily divided our population in low-FGD3-expressing tumours (undetectable staining or weak staining with ≤30% positive cells) and high-FGD3-expressing tumours (all other cases: strong or moderate staining, or weak staining with >30% positive cells). The level of significance decreased or was not achieved if we considered only the percentage of positive cells, with different cut-off points, or the staining intensity.

### Statistical analysis

The frequency distribution was assessed by Fisher’s Exact Test or Chi-Square Test, as appropriate. Chi Square Test for Trend was used to analyse the linear increasing trend of FGD3 staining intensity between two subgroups based on lymph node involvement. Disease-free interval and overall survival were calculated according to Kaplan-Meier method and evaluated by Log-Rank Test. Univariate Cox proportional hazards regression analysis was used to evaluate the effect of each prognostic factor on disease-free survival and overall survival. We used a multivariate Cox proportional hazards regression analysis, with forward selection of variables, to assess the independence of each prognostic variable. Data analysis was performed using IBM SPSS Statistics, version 17.0.

### Ethical approval

All procedures performed in this retrospective study involving human participants were in accordance with the ethical standards of the institutional research committee and with the 1964 Helsinki declaration and its later amendments or comparable ethical standards. This article does not contain any studies with animals performed by any of the authors.

### Informed consent

Informed consent was obtained from all individual participants included in the study.

## Data Availability

The datasets during and/or analysed during the current study are available from the corresponding author on reasonable request.
